# Farm Monitoring System with Drones and Optical Camera Communication

**DOI:** 10.3390/s24186146

**Published:** 2024-09-23

**Authors:** Shinnosuke Kondo, Naoto Yoshimoto, Yu Nakayama

**Affiliations:** 1 Department of Electrical Engineering and Computer Science, Tokyo University of Agriculture and Technology, Tokyo 184-8588, Japan; shinnosuke.kondo@ynlb.org; 2 Department of Opto-Electronics System Engineering, Chitose Institute of Science and Technology, Chitose 066-8655, Japan; n-yoshi@photon.chitose.ac.jp

**Keywords:** sensor network, optical camera communication, drone, LED, point-to-multipoint communication

## Abstract

Drones have been attracting significant attention in the field of agriculture. They can be used for various tasks such as spraying pesticides, monitoring pests, and assessing crop growth. Sensors are also widely used in agriculture to monitor environmental parameters such as soil moisture and temperature. Due to the high cost of communication infrastructure and radio-wave modules, the adoption of high-density sensing systems in agriculture is limited. To address this issue, we propose an agricultural sensor network system using drones and Optical Camera Communication (OCC). The idea is to transmit sensor data from LED panels mounted on sensor nodes and receive the data using a drone-mounted camera. This enables high-density sensing at low cost and can be deployed in areas with underdeveloped infrastructure and radio silence. We propose a trajectory control algorithm for the receiving drone to efficiently collect the sensor data. From computer simulations, we confirmed that the proposed algorithm reduces total flight time by 30% compared to a shortest-path algorithm. We also conducted a preliminary experiment at a leaf mustard farm in Kamitonda-cho, Wakayama, Japan, to demonstrate the effectiveness of the proposed system. We collected 5178 images of LED panels with a drone-mounted camera to train YOLOv5 for object detection. With simple On–Off Keying (OOK) modulation, we achieved sufficiently low bit error rates (BERs) under $10^{-3}$ in the real-world environment. The experimental results show that the proposed system is applicable for drone-based sensor data collection in agriculture.

## 1. Introduction

Agriculture is the cornerstone of human civilization, not only as the primary source of food but also as a provider of essential raw materials for numerous industries. It plays a crucial role in the economic development of nations, contributing significantly to their growth and stability [1,2]. Despite this, a significant proportion of farmers worldwide continue to rely on traditional farming methods that often result in suboptimal yields. To address these challenges, the concept of smart agriculture, which aims to enhance productivity and efficiency through the automation of agricultural practices, has gained significant attention [3].

Smart agriculture refers to the integration of advanced technologies, such as robotics, artificial intelligence (AI), and the Internet of Things (IoT), into farming practices. These technologies enable the optimization of various agricultural processes, leading to enhanced efficiency and productivity. Key elements of smart agriculture include the deployment of autonomous ground vehicles, drones, and agricultural sensors that provide real-time data for precision farming.

In recent years, the use of drones in smart farming systems has become increasingly widespread [4,5]. Agricultural drones can follow preprogrammed flight paths autonomously, reducing the need for manual intervention. These drones are capable of performing critical agricultural tasks both during the day and at night, thereby alleviating the workload of farmers [6]. Drones are used for a variety of agricultural applications, including the spraying of pesticides and fertilizers as well as pest monitoring. One of the most valuable functions of drones is their ability to capture data through multispectral cameras, which can assess crop health, detect early signs of disease, and predict optimal harvest times [7,8]. This technology allows farmers to remotely monitor the condition of their crops and take timely action to address potential issues. The continuous development of additional functionalities for drones holds great potential for further enhancing agricultural practices.

Environmental monitoring using sensors to measure parameters such as temperature, humidity, soil moisture, and light intensity is a critical component of smart agriculture. These sensors enable precise management of crop growth, allowing farmers to make data-driven decisions to optimize agricultural productivity. The collection of sensor data is typically achieved through wireless communication, with protocols such as Low-Power Wide-Area Networks (LPWANs) being widely adopted due to their efficiency in transmitting data over long distances. However, despite the relatively low cost of sensors, the communication modules necessary for radio-based data transmission are often prohibitively expensive. This presents a significant challenge for the deployment of high-density sensor networks, where a large number of sensors are required to comprehensively monitor agricultural environments. The high cost of such infrastructure limits the scalability of sensor-based systems, particularly in large or resource-constrained farming operations. In addition to the cost barrier, geographical and infrastructural challenges also hinder the adoption of wireless sensor networks. In mountainous or remote regions, where communication infrastructure is sparse or nonexistent, radio-based transmission may fall outside the coverage area, further complicating the implementation of sensor networks in smart agriculture. Addressing these infrastructural gaps is essential to fully realize the potential of sensor-driven technologies in agricultural management.

To address this issue, we propose an agricultural sensor network system that leverages Optical Camera Communication (OCC) technology. OCC is a communication method that uses visible light to transmit data between a light source and a camera. The idea behind the proposed system is to transmit sensor data from LED panels mounted on sensor nodes and receive the data using a drone-mounted camera. The proposed system offers high-density sensing at a low cost due to the use of LED modules, which significantly reduces the cost of wireless communication. Thanks to the use of visible light, OCC does not require a license for operation, making it a cost-effective alternative to traditional radio-based communication systems. To efficiently collect densely distributed sensor data, we propose a trajectory control algorithm for the receiving drone. The proposed algorithm calculates the flight route and speed based on the locations and sizes of the light sources and the specifications of the receiver camera.

The primary contribution of this paper is the development of a low-cost, high-density sensing system utilizing existing agricultural drones. The proposed system leverages OCC technology to enable efficient data collection from sensor nodes distributed across farmland. This system is particularly suitable for deployment in areas with limited infrastructure or radio communication constraints. We present preliminary experimental results from a leaf mustard farm in Kamitonda-cho, Wakayama, Japan, demonstrating the feasibility of the proposed system. We implemented a system where drones automatically collect data from sensor nodes distributed across farmland. An Arduino UNO microcontroller was used to manage the sensor nodes, and a DJI Mavic 2 Pro drone served as the data receiver. The experimental results confirmed that OCC-based data transmission was successful, with minimal errors, allowing for effective collection of sensor data.

The rest of this paper is organized as follows. Section 2 summarizes related research relevant to this study. In Section 3, we introduce the proposed system. We explain the overall flow of the proposed system and provide the flight route calculation method in the proposed algorithm. Then, Section 4 provides the performance of the proposed algorithm using computer simulations and the effectiveness of the algorithm. Experimental results are presented in Section 5. We implemented a program for the drone to move autonomously and evaluated the feasibility of the proposed system. Finally, Section 6 summarizes the conclusions of this paper.

## 2. Related Work

### 2.1. Smart Agriculture with Drones

Recent advancements in drone technology have enabled applications across many industries. In agriculture, drones have significantly improved efficiency and productivity [9]. Their high mobility and ability to collect large amounts of data make them essential tools for monitoring and data collection in farming [10,11]. Research on the use of drones in agriculture continues to grow [12]. Pasquale et al. [13] highlighted the innovative role of drones in agriculture. They demonstrated that drones can collect and analyze data with high precision, improving efficiency and productivity. Spoorthi et al. [14] proposed the use of drones for spraying pesticides and fertilizers. They found that drone-based spraying provides more uniform coverage and greater efficiency than traditional methods. Paulina et al. [15] studied crop condition monitoring using drones equipped with multispectral and temperature sensors. They calculated the Normalized Difference Vegetation Index (NDVI) through field tests. Namani et al. [16] developed a crop management system using real-time drone data, IoT, and cloud computing. This system optimizes irrigation by analyzing sensor data to provide water at the right time and in the required amounts. Jawad et al. [17] tackled the problem of limited drone flight time by developing wireless power transfer (WPT) technologies. Shidrokh et al. [18] proposed a routing algorithm for efficient and accurate data collection. This algorithm considers both flight time and speed. Ciciouglu et al. [19] created a drone-based system for monitoring large cornfields. Their drones, equipped with high-resolution cameras and sensors, allowed for real-time monitoring and efficient management of the fields. Moribe et al. [20] introduced a communication protocol for drones to monitor leaf temperature data using infrared thermometers. Their system combines wireless sensor networks with drones. This setup allows for real-time monitoring of large areas and rapid data collection. Drones in this system extend the communication range of sensor networks and improve crop yield monitoring.

### 2.2. Sensor-Based Monitoring in Agriculture

Sensor-based monitoring is considered one of the most important technologies for smart agriculture [21]. Ramson et al. [22] developed a real-time soil health monitoring system based on a Long-Range Wide-Area Network (LoRaWAN). In this system, data collected from soil sensors are transmitted to a cloud platform via the LoRaWAN. Chen et al. [23] proposed a real-time temperature monitoring and frost prevention control system. This system, based on Z-BEE wireless sensor networks, automatically monitors temperature to prevent frost damage to tea plants. Khalid et al. [24] introduced an energy-efficient and secure IoT-based wireless sensor network framework for smart agriculture. Their framework provided stable network performance between agricultural sensors and ensured secure data transmission. Valecce et al. [25] evaluated the performance of data collection and monitoring systems in agriculture using Narrowband Internet of Things (NB-IoT) technology. They analyzed performance metrics such as data accuracy, communication latency, and battery consumption. Tsai et al. [26] proposed an environmental monitoring system for microfarms. In this system, environmental data collected from sensors are transmitted to a cloud server via a Wi-Fi network.

### 2.3. Optical Camera Communication (OCC)

OCC is a technology that utilizes a camera and a light source for data transmission. Light sources such as LED panels, displays, and digital signage are used as transmitters to send digital data in the form of optical signals [27]. A receiver camera captures the transmitted signals using a complementary metal-oxide-semiconductor (CMOS) sensor. OCC is particularly suited for point-to-multipoint (P2MP) communication, as it can use multiple light sources in a given area with minimal interference between transmitters. The multiple light signals captured in the image can be demodulated by cropping the pixels corresponding to each light source [28]. OCC falls under the category of visible light communication (VLC), where a light source serves as the transmitter and a camera functions as the receiver.

The primary advantages of OCC include cost efficiency, unlicensed operation, and low power consumption. Since most modern agricultural drones are equipped with cameras [8], they can serve as effective receivers. This makes OCC a promising technology for remote sensing applications in agriculture. In [29], the performance of OCC was evaluated based on parameters such as camera sampling rate, exposure time, focal length, pixel edge length, transmitter configurations, and optical flicker rate. The study defined the signal-to-interference-plus-noise ratio (SINR) and analyzed different modulation schemes. Ambient light interference is a major challenge for visible light communication, including OCC. Some studies have proposed solutions to mitigate this issue. Islim et al. [30] examined the impact of sunlight on VLC. They clarified the specific effects of sunlight and proposed a system to counteract them, achieving data rates exceeding 1 Gb/s even under strong sunlight. Takai et al. [31] developed a vehicle-to-vehicle communication system using OCC. They achieved a reception rate of 13.0 frames per second (fps) under real-world driving and outdoor lighting conditions. Elizabeth et al. [32] conducted experiments evaluating a 400-m communication link using OCC in outdoor environments. Their results demonstrated stable communication performance under various weather and lighting conditions. Drone-based OCC systems have also been studied in various applications. Takano et al. [33] investigated OCC systems using drones equipped with RGB-LEDs and a high-speed camera to achieve communication over a distance of 300 m. Their experimental results demonstrated that efficient and accurate communication is possible by employing object detection techniques. Li et al. [34] focused on the trajectory control of drones to maintain line-of-sight (LoS) communication links in OCC systems. They proposed a distributed trajectory control algorithm that allows drones to avoid inter-light interference during communication. However, the previous studies did not consider dense sensor data collection in agriculture using drones and OCC.

Furthermore, to the best of our knowledge, there has been no previous work on drone-based agricultural sensor networks using OCC. This paper proposes a novel system that combines drones and OCC for high-density sensor data collection in agriculture. The proposed system leverages the advantages of OCC to enable efficient data transmission from sensor nodes to a drone-mounted camera. The system is designed to be cost-effective, scalable, and suitable for deployment in areas with limited infrastructure. The details of the proposed system are presented in the following section.

## 3. Proposed Scheme

### 3.1. Concept

**Figure 1:**
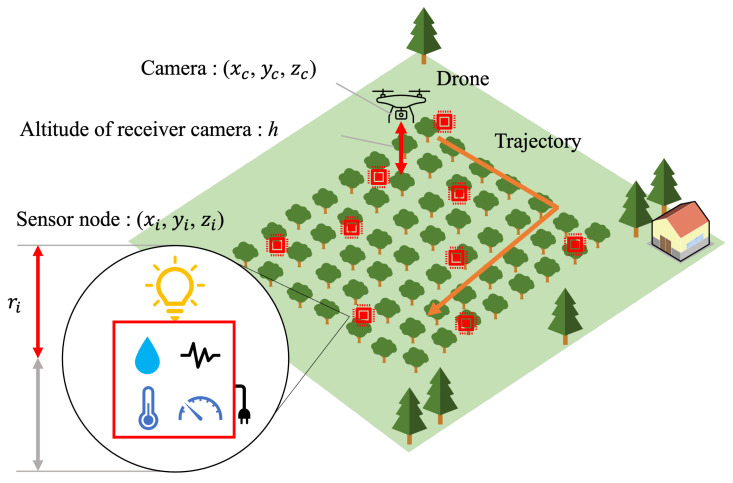
Conceptual system architecture (Orange arrows: drone trajectory).

**Figure 2:**
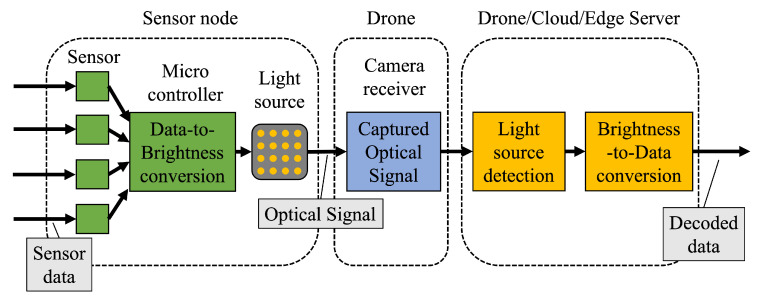
Block diagram of proposed system.

We propose a drone-based sensor network system using OCC. The conceptual system architecture is illustrated in Figure 1, while the block diagram of the system is detailed in Figure 2. Many sensor nodes are deployed in a target area. A sensor node is equipped with sensors and a light source. The light source can be an LED light, an LED panel, or a display. The sensor data are encoded and modulated as optical signals to be transmitted from the light source. A receiver drone equipped with a camera moves around the target area to film the optical signals from the sensor nodes. The received signals are demodulated at either the drone or a cloud/edge server in real time.

We propose a trajectory control algorithm for the receiver drone to efficiently collect the sensor data. The proposed algorithm calculates the flight route and speed based on the locations and sizes of the light sources and the specifications of the receiver camera. The proposed system can accommodate a large number of sensor nodes without time- and frequency-domain interference thanks to OCC. It can be deployed in radio quiet or infrastructure-underdeveloped areas since it does not use any radio waves.

### 3.2. Variable Definition

#### 3.2.1. Coordination Systems

In this section, we describe the coordination systems used in the proposed model. We define the global coordination system as (x,y,z). The camera coordination system is also defined as (X,Y,Z). The *Y*-axis is aligned with the center line of the image. Note that the origin is set to the receiver camera at $\left(x_{c}\left(t\right),y_{c}\left(t\right),z_{c}\left(t\right)\right)$. The elevation angle of the camera is defined as θ.

#### 3.2.2. Variables

The variables used in the proposed model are summarized in Table 1. Let I denote the set of sensor nodes and i,j denote the identifier for them. The position of the *i*th sensor node in the global coordinate system is defined as $\left(x_{i},y_{i},z_{i}\right)$. For simplicity, a sensor node is approximated as a sphere with radius $r_{i}$. Let $\left(u_{i},v_{i}\right)$ denote the coordinates of the center of the *i*th sensor node in the image plane. The horizontal and vertical resolutions of the image are $l_{h}$ and $l_{v}$, respectively. The horizontal and vertical angles of view of the camera are denoted as $\phi_{h}$ and $\phi_{v}$. The focal length of the camera is described as *f*. The size of the image sensor is defined as ρ.

### 3.3. System Model

Based on the defined variables, we present the system model that describes how the sensor data are collected by the receiver drone.

#### 3.3.1. Coordination Transformation

The positions of the sensor nodes in the image plane are computed with the coordination transformation. Since we assume a moving receiver drone, the relative position between $\left(x_{i},y_{i},z_{i}\right)$ and the origin $\left(x_{c}\left(t\right),y_{c}\left(t\right),z_{c}\left(t\right)\right)$ is computed. The position of the *i*th sensor node in the camera coordination system is formulated as

(1) $$\left[\begin{array}{c} X_{i}\left(t\right)\\ Y_{i}\left(t\right)\\ Z_{i}\left(t\right) \end{array}\right]=\left[\begin{array}{ccc} 1 & 0 & 0\\ 0 & \cos(-\theta ) & -\sin(-\theta )\\ 0 & \sin(-\theta ) & \cos(-\theta ) \end{array}\right]\left[\begin{array}{c} x_{i}-x_{c}\left(t\right)\\ y_{i}-y_{c}\left(t\right)\\ z_{i}-z_{c}\left(t\right) \end{array}\right]$$

which consists of the parallel displacement and the rotation by the elevation angle.

Then, the position of the *i*th sensor node in the image plane is computed with the perspective transformation as

(2) $$\left(u_{i}\left(t\right),v_{i}\left(t\right)\right)=\left(\begin{array}{cc} \frac{l_{h}}{2\tan\left(\frac{\phi_{h}}{2}\right)} & 0\\ 0 & \frac{l_{v}}{2\tan\left(\frac{\phi_{v}}{2}\right)} \end{array}\right)\left(\begin{array}{c} \frac{X_{i}\left(t\right)}{Y_{i}\left(t\right)}\\ \frac{Z_{i}\left(t\right)}{Y_{i}\left(t\right)} \end{array}\right)+\left(\begin{array}{c} \frac{l_{h}}{2}\\ \frac{l_{v}}{2} \end{array}\right).$$

**Figure 3:**
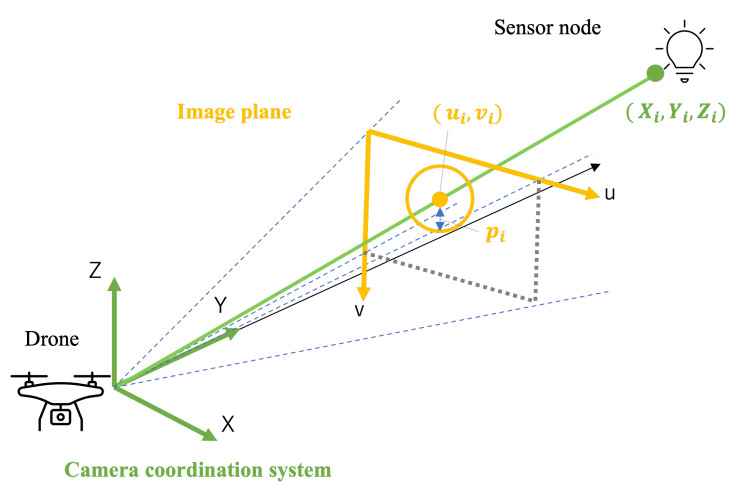
Perspective transformation to image plane.

The perspective transformation is depicted in Figure 3. Let $p_{i}$ denote the size of a sensor node in the image plane. It is calculated with the radius of the projected circle as

(3) $$p_{i}=\frac{r_{i}}{\rho Y_{i}\left(t\right)}f.$$

The conditional expression that the *i*th sensor node is filmed by the receiver drone is formulated as

(4) $$\begin{array}{c} \left\{\begin{array}{c} 0\leq u_{i}\left(t\right)-p_{i}\wedge u_{i}\left(t\right)+p_{i}\leq l_{h}\\ 0\leq v_{i}\left(t\right)-p_{i}\wedge v_{i}\left(t\right)+p_{i}\leq l_{v}. \end{array}\right. \end{array}$$

#### 3.3.2. Ground Coverage

**Figure 4:**
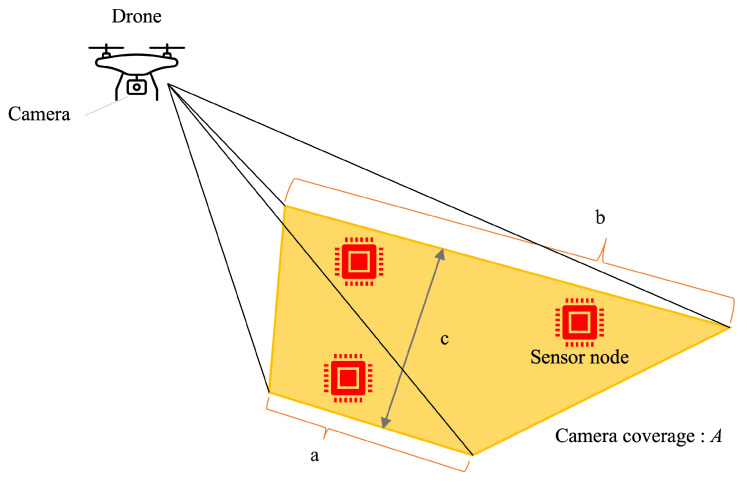
Ground coverage of drone-mounted camera (a: top length, b: bottom length, c: height of trapezoid).

The ground coverage of the drone-mounted camera is a trapezoid A shown in Figure 4. The ground coverage of this trapezoid is defined as

(5) $$\begin{array}{c} \left\{\begin{array}{c} a=\frac{2h\cos\frac{\phi_{v}}{2}\tan\frac{\phi_{h}}{2}}{\cos\left(\theta -\frac{\phi_{v}}{2}\right)}\\ b=\frac{4h\tan\frac{\phi_{h}}{2}}{\cos\theta }-a\\ c=h\left\{\tan\left(\theta +\frac{\phi_{v}}{2}\right)-\tan\left(\theta -\frac{\phi_{v}}{2}\right)\right\} \end{array}\right. \end{array}$$

where *a* is the top length, *b* is the bottom length, *c* is the height of the trapezoid A, and *h* is the altitude of the receiver camera. The receiver can simultaneously receive the optical signals from the sensor nodes within this trapezoid at the same time. These sensor nodes are grouped as a single virtual sensor node. The sensors within a trapezoid are grouped to improve the efficiency of data collection. Since the size of the trapezoid increases in accordance with the altitude *h*, it is efficient to maximize *h*.

#### 3.3.3. Altitude Limit

As the altitude of the drone-mounted camera increases, the size of a sensor node in the image plane decreases, and the accuracy of signal demodulation decreases. The altitude of the receiver camera is limited to establish a stable link with a ground sensor node. The constraint is described as

(6) $$p_{i}\geq p_{th},$$

where $p_{th}$ denotes the threshold for the size of a sensor node in the image plane. By satisfying (6), the optical signal transmitted from the sensor node can be correctly demodulated. In other words, a light source must be sufficiently large in the image to correctly demodulate the optical signals. From (3) and (6), the altitude limit of a receiver camera is determined as

(7) $$h\leq \frac{fr_{i}}{\rho p_{th}}\cos\theta .$$

#### 3.3.4. Transmission Time

Here we formulate the data transmission time from a sensor node. The transmission rate of an OCC link is determined by the modulation number and symbol rate. Optical spatial modulation and color-shift keying (CSK) are employed to increase the data rate. Multiple light sources are employed in optical spatial modulation. CSK exploits the design of three-color luminaires of LED. Optical signals are modulated by modifying the light intensity to generate predefined constellation symbols [35]. The range of the symbol rate is constrained by the frame rate of the receiver camera and the image processing speed.

The transmission rate is formulated as

(8) $$R_{i}=S_{i}D\log_{2}N_{i},$$

where $R_{i}$ is the data rate, $S_{i}$ is the optical spatial multiplicity, *D* is the symbol rate, and $N_{i}$ is the number of constellation symbols. The maximum transmission time is computed as

(9) $$T_{i}=\frac{M_{i}}{R_{i}},$$

where we define the maximum data size as $M_{i}$. This transmission time has a direct impact on the trajectory of the drone, as it must remain within a sufficient time range to complete the data reception.

#### 3.3.5. Trajectory Requirement

It is required for the receiver drone to film each sensor node for sufficient time duration to receive the transmitted data. Let $\tau_{i}$ denote the time length where (5) is satisfied. To ensure receiving the maximum data size from the *i*th sensor node, $\tau_{i}$ must satisfy

(10) $$\tau_{i}\geq T_{i}.$$

The time required to receive the maximum data size from a single virtual sensor node can be formulated as

(11) $$\tau_{i}\geq \frac{M_{i}}{S_{i}D\log_{2}N_{i}}.$$

The trajectory of the receiver drone must be determined to satisfy (11).

### 3.4. Algorithm

**Figure 5:**
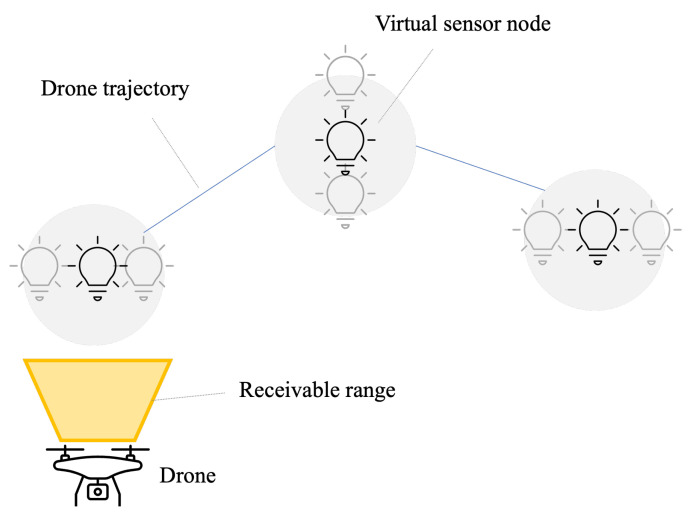
Concept of trajectory control algorithm.

The goal of the proposed algorithm is to achieve the approximately shortest trajectory ensuring data transmission from all sensor nodes. The concept of the trajectory control algorithm is shown in Figure 5. The proposed algorithm is summarized in Algorithm 1. The proposed algorithm consists of the following steps.

#### 3.4.1. Node Clustering

The sensor nodes are organized into clusters to form virtual sensor nodes. Neighboring sensor nodes are grouped to satisfy (5). V represents a set of sensor nodes I that have been grouped using an appropriate clustering algorithm. It is verified that all sensor nodes in the *v*th group, $V_{v}$, are situated within the trapezoid A, thereby satisfying (5). If any sensor node within $V_{v}$ does not reside within A, clustering is repeated. This guarantees that the groups satisfy (5). The clustering algorithm is selected based on the distribution of sensor nodes. The altitude of the receiver drone, *h*, is set to the highest value that satisfies (7). Minimizing the number of virtual sensor nodes reduces the receiver drone’s flight time.
**Algorithm 1** Trajectory control algorithm.**Input:** I**Output:** Trajectory, Times  1:# Node clustering  2:**function** IsInTrapezoid($V_{v}$)  3:    **for** $i\in V_{v}$ **do**  4:        **if** $(x_{i},y_{i},z_{i})\notin A$ **then**  5:           **return** False  6:        **end if**  7:    **end for**  8:    **return** True  9:**end function**10:**function** ClusterNodes(I)11:    V← AnyClusteringAlgorism(I)12:    clusters←*∅*13:    **for** *v* in range(|V|) **do**14:        **if** $IsInTrapezoid(V_{v})=TRUE$ **then**15:           $clusters.append(V_{v})$16:        **else**17:           clusters.append(ClusterNodes($V_{v}$))18:        **end if**19:    **end for**20:    **return** clusters21:**end function**22:**function** CalculateFilmingTime(clusters)23:    times←*∅*24:    **for** c∈clusters **do**25:        $\tau_{max}$← 026:        **for** i∈c **do**27:           **if** $\tau_{i}>\tau_{max}$ **then**28:               $\tau_{max}$←$\tau_{i}$29:           **end if**30:        **end for**31:        $times.append(\tau_{max})$32:    **end for**33:    **return** times34:**end function**35:clusters← ClusterNodes(I)36:# Graph generation37:G← GenerateGraph(clusters)38:# Trajectory determination39:Trajectory← TSP(G)40:Times← CalculateFilmingTime(clusters)

#### 3.4.2. Graph Generation

Following the clustering of the sensor nodes, a graph representing the virtual sensor nodes is constructed. This graph, denoted by G=(V,E), comprises the set of virtual sensor nodes V and the edges E. The graph enables the computation of an optimized trajectory using a traveling salesman problem (TSP)-based approach.

#### 3.4.3. Trajectory Determination

The trajectory is determined by solving a TSP on graph G. The time required for filming each virtual node is calculated in accordance with the formula presented in (9). To ensure reliable data transmission, the drone allocates sufficient time to film each sensor node. This duration is calculated using (11), which determines the required filming time based on sensor data size and transmission rate. Meeting this condition guarantees successful reception of all data from each sensor node. Consequently, the receiver drone can efficiently collect data from all sensor nodes in the farm.

## 4. Computer Simulation

The performance of the proposed algorithm was verified via computer simulation.

### 4.1. Simulation Conditions

Table 2 summarizes the parameters used in the simulation. To evaluate the performance of the proposed algorithm under realistic conditions, the parameters are the same as those used in the preliminary experiments in Section 5. These values are based on the specifications of the drone and the sensor nodes. The altitude of the receiver camera was set to 5 m. The speed of the drone was set to 3 m/s. The drone stops to film the sensor node for a certain amount of time depending on the size of the transmitted data. The filming time ranged from 0 to 3 s. The horizontal and vertical angles of view were $77^{{}^{\circ}}$ and $40^{{}^{\circ}}$, respectively. The elevation angle of the camera was set to $20^{{}^{\circ}}$.

The sensor nodes were distributed randomly in a square area. The density of sensor nodes was set to 4 per 10×10 m. The size of the study area ranged from 50×50 to 200×200 m. The simulation was iterated 1000 times for each condition with different seeds. The performance of the proposed trajectory control algorithm was compared with that of the shortest path algorithm. We employed several clustering algorithms such as k-means, group average method, and Ward’s method.

### 4.2. Simulation Results

#### 4.2.1. Total Trajectory Length

**Figure 6:**
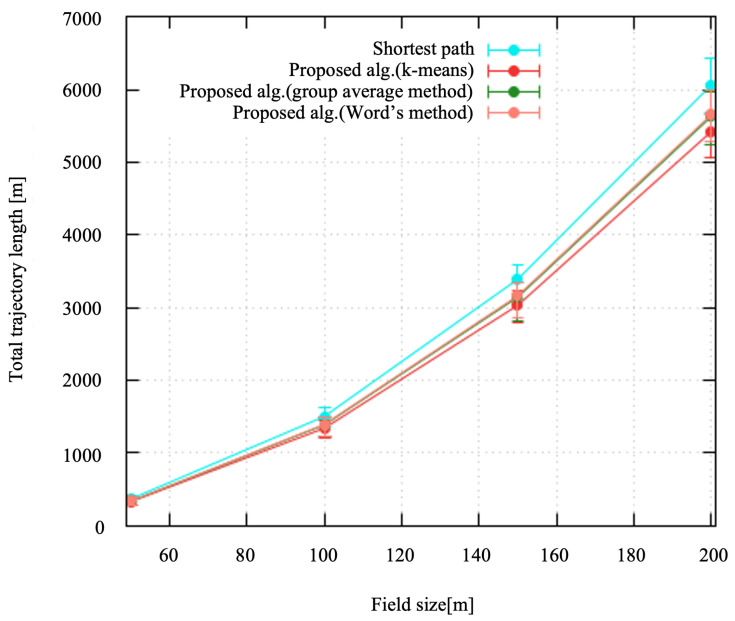
Total trajectory length.

Figure 6 shows the total trajectory length of the drone to collect the data from all of the deployed sensor nodes. The points represent the average. The error bars show the maximum and minimum values. The trajectory length increased according to the field size. As the field size increased beyond 120 m, a clear separation in performance became more apparent. The shortest path consistently resulted in the longest trajectory across all field sizes, whereas the proposed algorithm demonstrated slightly more efficient trajectory lengths. The proposed algorithm using k-means tended to yield the shortest trajectory, closely followed by the group average method and Ward’s method. This suggests that the proposed method is more efficient in optimizing the total trajectory length in larger fields.

#### 4.2.2. Total Travel Time

**Figure 7:**
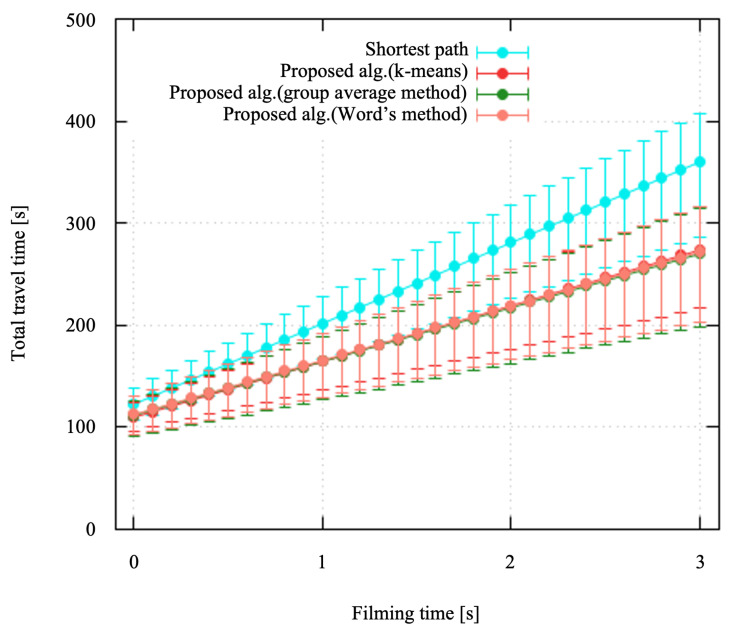
Total travel time.

Figure 7 shows the total travel time to receive all the data from the sensor nodes where the field size was 50×50 m. The points and error bars represent the average and the maximum/minimum values. The total travel time increased as the filming time, i.e., data size of the sensor nodes, increased. As the filming time increased from 0 to 3 s, the total travel time for all algorithms increased proportionally. The shortest path consistently produced the highest total travel time over the entire range of filming times. On the other hand, the proposed algorithm achieved significantly lower total travel times. This is because the sensor nodes were clustered so that the receiver drone simultaneously received data from multiple sensor nodes. Among the clustering algorithms, the k-means algorithm typically showed the lowest total travel time, closely followed by the group average and Ward’s methods. We confirmed that the proposed algorithm outperformed the shortest path algorithm regardless of the conditions and clustering algorithms. Although the expected performance was almost the same with the clustering algorithms employed, the minimum value of the total travel time was slightly shorter with the group average method.

## 5. Experimental Results

This section presents the experimental results of the proposed scheme. The feasibility of the proposed system and the accuracy of data transmission in the real environment were confirmed using a drone and an implemented sensor node.

### 5.1. Experimental Condition

#### 5.1.1. Overview

**Figure 8:**
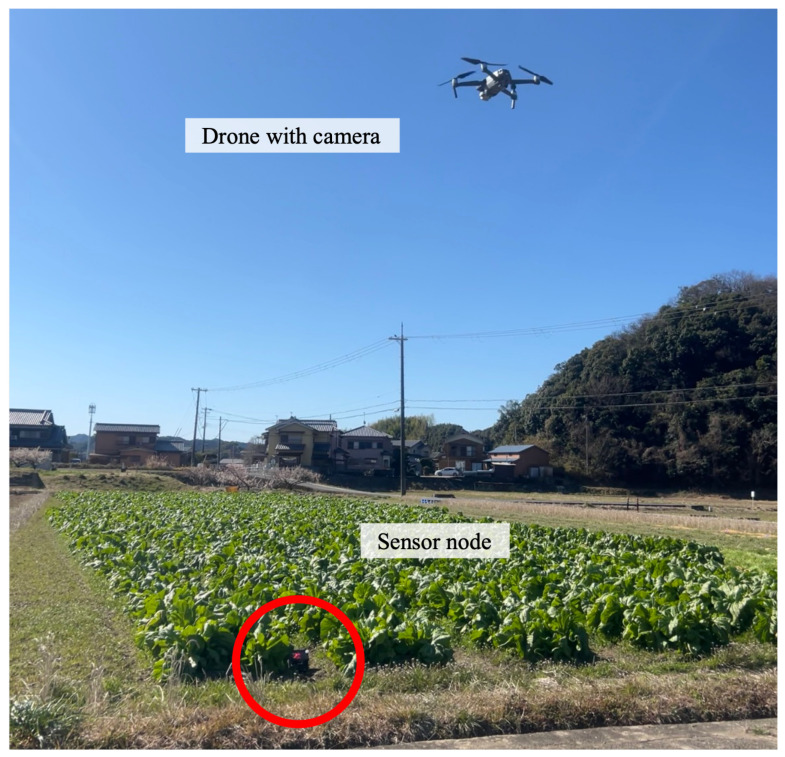
Experimental setup.

The experiments consisted of three steps. First, the ground coverage of the drone-mounted camera was confirmed in the real environment. The theoretical equations were verified under different elevation angles of the camera. Second, an object detection model was trained using YOLOv5 in the real-world environment and evaluated for accuracy. This model is used in OCC to detect LED panels in images. Third, the accuracy of data transmission of OCC in the real-world environment was evaluated based on the bit error rate (BER). The performance was evaluated under different daylight conditions. Figure 8 shows the experimental setup. The deployed sensor nodes are marked with circles. The sensor nodes transmit sensor data.

#### 5.1.2. Experimental Setup

We employed a Mavic 2 Pro drone, launched by DJI Co., Ltd. Shenzhen, China The resolution of the receiving camera was 1080×1920, the pixel count was 2 mega pixels, and the frame rate was 30 fps. The focal length was f=28 mm, and the zoom magnification was set to M=1.

**Figure 9:**
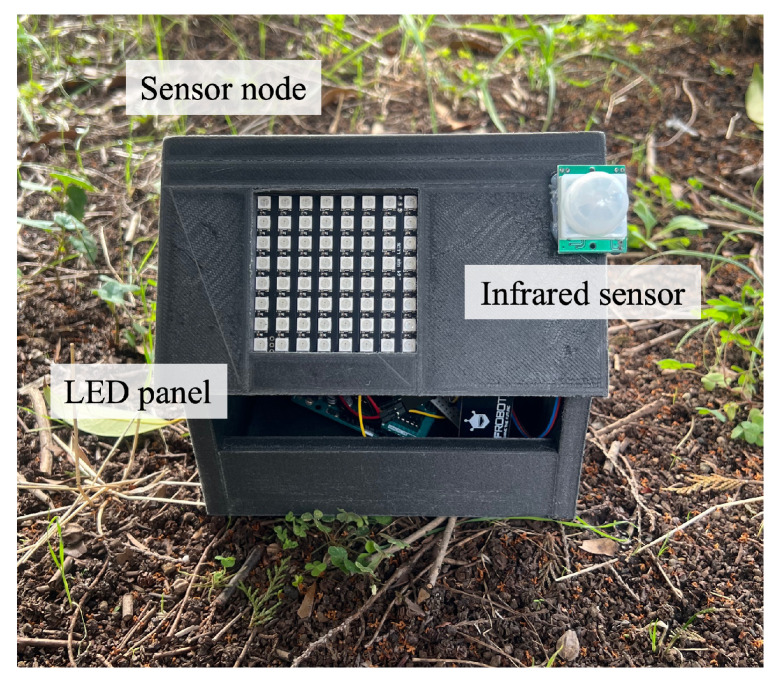
Sensor node.

Figure 9 shows a sensor node. A sensor node was equipped with five sensors and an LED panel. It collected temperature, humidity, illumination, soil water content, and infrared sensor data. We employed WS2812B serial LED panels with 64 LED lights arranged in a square. An Arduino UNO microcontroller was connected to the sensors and the LED panel to control them.

The battery supplies power to the sensors, the Arduino UNO microcontroller, and the LED panel.

**Figure 10:**
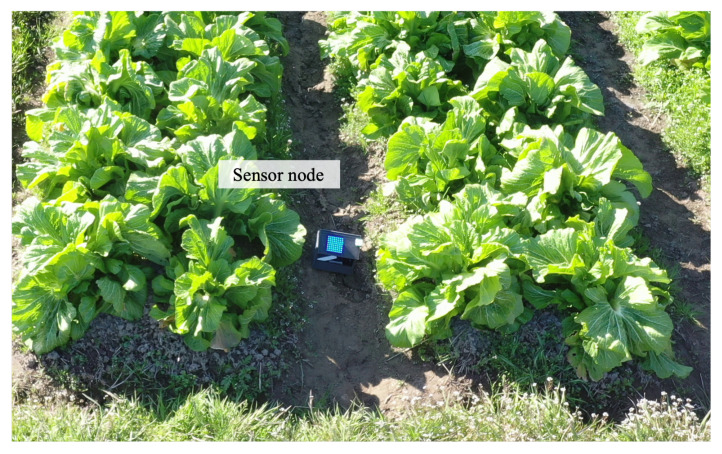
Sensor node taken from drone.

In this experiment, On–Off Keying (OOK) was used as the modulation scheme. The Arduino UNO microcontroller encoded and modulated the sensor data as optical signals to be transmitted by the LED panel. A sensor node transmitted optical signals when a drone flew over it by detecting the drone with an infrared sensor to reduce energy consumption. An example image of the sensor node taken by the drone is shown in Figure 10.

**Figure 11:**
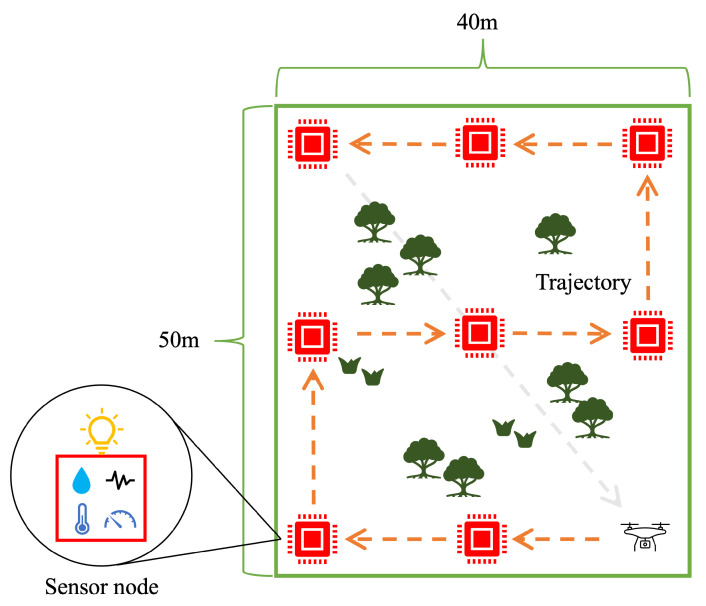
Sensor node placement (Red point: sensor node, Orange arrow: drone trajectory).

The study area was a leaf mustard farm in Kamitonda-cho, Wakayama, Japan. We deployed eight sensor nodes in an area of 50×40 m^2^, which is shown in Figure 11. The flight trajectory of the drone was calculated using the proposed algorithm.

#### 5.1.3. Coding and Modulation

In this experiment, the sensor data collected at the sensor nodes were encoded and modulated as optical using On–Off Keying (OOK). More specifically, the sensor data were converted to binary. The bit sequence was then converted into a data signal using 3b4b coding. The LED panels mounted on the sensor nodes were used as transmitters. A signal sent from a data panel carried one bit with OOK. In other words, the one bit was modulated as the on–off signals of the blue color.

#### 5.1.4. Demodulation

The transmitters mounted on the sensor nodes transmitted continuous light that was captured by a drone-mounted camera. The captured video was sent to an edge server to be divided into a series of static images. The edge server used the filmed images to demodulate the optical signals from the sensor nodes. Specifically, it demodulated the optical signals by mapping the RGB values of the LED panels to the (x, y) color space. The color coordinates of each bit were determined by pilot signals. The optical signals were demodulated based on the determined color coordinates.

### 5.2. Results

#### 5.2.1. Model Confirmation

First, we confirmed the mathematical model based on the coordination transformation. We measured the ground coverage of the drone-mounted camera in the real environment. Then, we compared the measured results with the theoretical model formulated in (5). Table 3 summarizes the difference of the measured values from the theoretical values. From this result, we confirmed the feasibility of the mathematical model.

#### 5.2.2. Light Source Detection

We employed YOLOv5, which is a famous machine learning model using a CNN, to detect the LED panels from the optical signal images. To ensure the recognition accuracy of the LED panels mounted on the sensor nodes, we collected 5178 images as training data and marked the positions of the LED panels. The resolution of the images was changed to 1920×1080. The results of the machine learning experiments with different parameters are presented in Table 4. YOLOv5l was selected as the model weight due to its superior detection accuracy and relatively short training time. We evaluated the performance of each parameter configuration by comparing the final loss values and the mean Average Precision (mAP). After comparing all configurations, we adopted the parameter set with a batch size of 16, an image size of 640, 300 epochs, and a final loss of 0.0030712, achieving an mAP of 0.71541, as indicated by the underlined values in Table 4. The PC environment was Ubuntu 20.04 and the GPU environment was NVIDIA GeForce RTX 3090. The software environments were CUDA 11.3, CUDNN 8.8, and Python 3.8. The datasets were obtained by changing the location and time at which the images were taken to improve recognition accuracy under different conditions. This is because noise from ambient light changes the visibility of LED panels.

**Figure 12:**
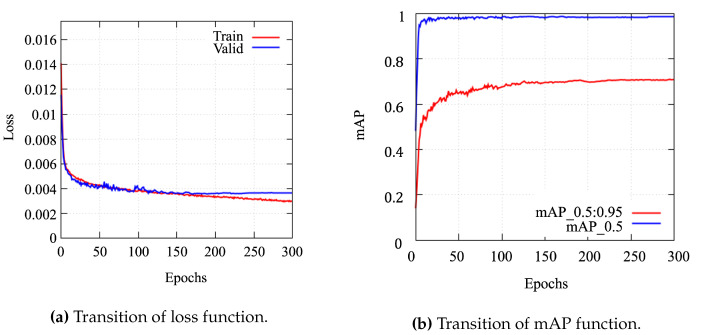
Recognition accuracy of the LED panels.

The results for the detection accuracy of the LED panels using YOLOv5 are shown in Figure 12. The accuracy of the object detection model was evaluated using loss and mAP. The object loss function is a measure of the probability that the detection target is within the region of interest. The lower the value of the loss function, the higher the accuracy. Figure 12a shows the evolution of the loss function per epoch. Before the training batch reached 50, both loss function values decreased rapidly. When the training batch reached 50, the decrease in both loss function values gradually slowed down. The mAP is the average of the average accuracy per class. In this paper, mAP and AP are equal because the number of classes is one. The higher the mAP value, the more accurate the network. Figure 12b shows the evolution of mAP per epoch. The value of mAP_0.5 reached 0.98. The mAP_0.5:0.95 reached 0.71. We confirmed that the object detection model successfully detected the LED panels.

#### 5.2.3. Signal Reception

**Figure 13:**
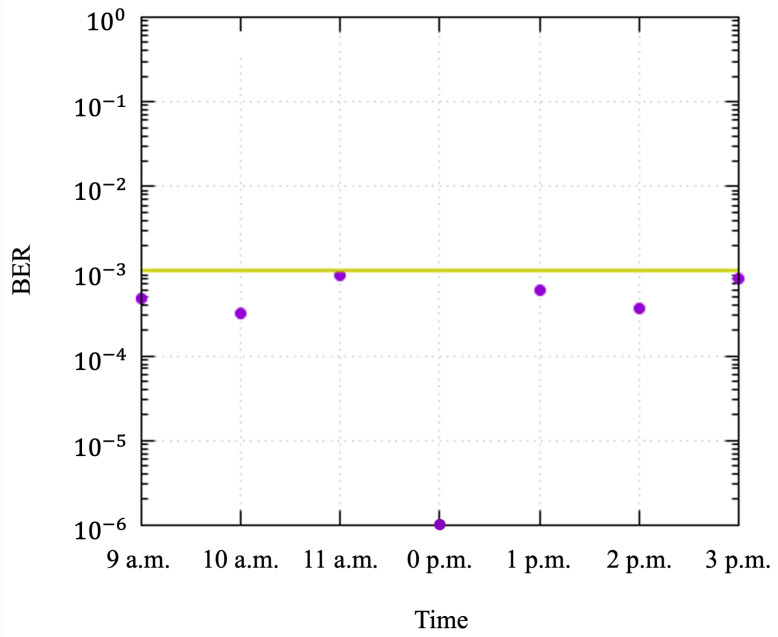
Bit error rate with threshold of $1\times 10^{-3}$.

First, we confirmed that the drone had successfully filmed all the sensor nodes for a sufficient duration. The drone autonomously moved along the calculated trajectory to film the optical signals from the LED panels. The sensor nodes detected the drone and transmitted sensor data. Second, we measured the bit error rate (BER) to evaluate the accuracy of data transmission in the real environment. To evaluate the performance under different outdoor lighting conditions, the BER was measured per filmed hour. The calculated BER is shown in Figure 13. The average BER measured in this experiment was 0.00043338. Assuming the use of 7% hard decision forward error correction (HD-FEC), the BER threshold was set to $1\times 10^{-3}$. A receiver drone equipped with a camera received the transmitted data with sufficiently few errors. A receiver drone equipped with a camera received the transmitted data without error. As a result, it was confirmed that the BER was low enough to receive the sensor data regardless of the observation time.

## 6. Conclusions

In this study, we introduced a sensor network system for farm monitoring that combines drones and OCC. The proposed system allows for high-density sensing at a reduced cost. By using OCC, the system collects data from multiple sensor nodes spread across agricultural fields. This approach minimizes the need for expensive communication infrastructure and addresses the challenge of deploying high-density sensor networks in agriculture. We developed a trajectory control algorithm to optimize data collection. This algorithm improves communication efficiency between drones and sensor nodes. It utilizes OCC’s strength in point-to-multipoint communication by reducing interference between multiple light sources. From computer simulations, we confirmed that the proposed algorithm reduces total flight time by 30% compared to a shortest-path algorithm. Our preliminary experiments at a leaf mustard farm in Kamitonda-cho, Wakayama, Japan, demonstrated the feasibility of the system. We collected 5178 images of LED panels with a drone-mounted camera to train YOLOv5 for object detection. With simple On–Off Keying (OOK) modulation, we achieved sufficiently low bit error rates (BERs) under $10^{-3}$ in the real-world environment. These results validate the system’s potential to enhance agricultural practices with a cost-effective and scalable sensor network.

The proposed system is a significant step toward promoting intelligent agriculture. It offers a practical solution for overcoming the challenges of cost and infrastructure. The system is highly adaptable and can be deployed in areas with limited infrastructure or without radio-based communication, making it suitable for diverse agricultural environments. A major benefit of the proposed system is its compatibility with existing agricultural drones, such as those used for spraying pesticides. These drones can be easily repurposed as receivers within the OCC communication framework. Additionally, the cameras used for OCC can provide visual crop monitoring, offering dual functionality for data collection and crop observation. Future work will focus on improving the system’s capabilities, particularly in integrating self-localization features to enhance drone navigation and control.

## Figures and Tables

**Table 1:** Variables.

Variable	Definition
I	Set of sensor nodes
i,j	Sensor node identifier in I
$r_{i}$	Radius of *i*th sensor node
$\left(x_{i},y_{i},z_{i}\right)$	Position of *i*th sensor node in global coordinate system
$\left(x_{c},y_{c},z_{c}\right)$	Position of camera in global coordinate system
$\left(u_{i},v_{i}\right)$	Position of *i*th sensor node in image plane
$l_{h}$	Horizontal resolution of image plane
$l_{v}$	Vertical resolution of image plane
$\phi_{h}$	Horizontal angle of view
$\phi_{v}$	Vertical angle of view
θ	Elevation angle of camera
*f*	Focal length of camera
ρ	Image sensor size
$R_{i}$	Data rate
$S_{i}$	Spatial multiplicity
*D*	Symbol rate
$N_{i}$	Symbol number for CSK
$M_{i}$	Maximum data size
$T_{i}$	Transmission time
*a*	Top length of camera coverage trapezoid
*b*	Bottom length of camera coverage trapezoid
*c*	Height of camera coverage trapezoid
*h*	Altitude of receiver camera
A	Ground coverage of receiver camera

**Table 2:** Simulation parameters.

Parameter	Value
Altitude of receiver camera *h*	5 m
Speed of drone	3 m/s
Horizontal angle of view $\phi_{h}$	$77^{{}^{\circ}}$
Vertical angle of view $\phi_{v}$	$40^{{}^{\circ}}$
Elevation angle of camera θ	$20^{{}^{\circ}}$

**Table 3:** Difference between measured and theoretical values [m].

θ [Deg]	*a*	*b*	*c*
0	0.070	0.070	0.012
30	0.080	0.044	0.015
45	0.009	0.010	0.018

**Table 4:** Detection accuracy per parameter.

Batch	Img-Size	Weight	Epochs	Loss	mAP_0.5:0.95
16	640	YOLOv5l	300	0.0030712	0.71541
16	320	YOLOv5l	300	0.0034124	0.70959
8	640	YOLOv5l	300	0.0033053	0.68308
8	320	YOLOv5l	300	0.0036273	0.68849

## Data Availability

The data presented in this study are available on request from the corresponding author.
